# Management of Salivary Gland Tumours: United Kingdom National Multidisciplinary Guidelines

**DOI:** 10.1017/S0022215116000566

**Published:** 2016-05

**Authors:** S Sood, M McGurk, F Vaz

**Affiliations:** 1 Department of Otolaryngology – Head and Neck Surgery, Bradford Teaching Hospitals, Bradford, UK; 2 Department of Oral and Maxillofacial Surgery, Guy's Hospital, London, UK; 3 Department of Head and Neck Surgery, University College London Hospital, London, UK

## Abstract

This is the official guideline endorsed by the specialty associations involved in the care of head and neck cancer patients in the UK. Salivary gland tumours are rare and have very wide histological heterogeneity, thus making it difficult to generate high level evidence. This paper provides recommendations on the assessment and management of patients with cancer originating from the salivary glands in the head and neck.

**Recommendations:**

• Ultrasound guided fine needle aspiration cytology is recommended for all salivary tumours and cytology should be reported by an expert histopathologist. (R)

• Adjuvant radiotherapy (RT) following surgery is recommended for all malignant submandibular tumours except in cases of small, low-grade tumours that have been completely excised. (R)

• For benign parotid tumours complete excision of the tumour should be performed and offers good cure rates. (R)

• In the event of intra-operative tumour spillage, most cases need long-term follow-up for clinical observation only. These should be raised in the multidisciplinary team to discuss the merits of adjuvant RT. (G)

• As a general principle, if the facial nerve function is normal pre-operatively then every attempt to preserve facial nerve function should be made during parotidectomy and if the facial nerve is divided intra-operatively then immediate microsurgical repair (with an interposition nerve graft if required) should be considered. (G)

• Neck dissection is recommended in all cases of malignant parotid tumours except for low-grade small tumours. (R)

• Where malignant parotid tumours lie in close proximity to the facial nerve there should be a low threshold for adjuvant RT. (G)

• Adjuvant RT should be considered in high grade or large tumours or in cases where there is incomplete or close resection margin. (R)

• Adjuvant RT should be prescribed on the basis of clinical factors in addition to histology and grade, e.g. stage, pre-operative facial weakness, positive margins, peri-neural invasion and extracapsular spread. (R)

## Introduction

Salivary gland malignancies are rare and the understanding of this disease is mostly based on clinical series rather than randomised evidence which is unlikely to emerge for these tumours. Approximately 300 cases are registered each year in England and Wales of which fewer than 10 occur in children (under 19 years of age).^1^ Population-based studies report that in a population of one million, eight to nine malignant salivary gland tumours can be expected per annum. Interspersed with this, malignant salivary gland disease is a larger workload of benign tumours, often performed by the non-head and neck oncological surgeon that also presents challenges from a management perspective.

Although, overall, tumours are more common in the parotid, the incidence of malignancy is higher in the submandibular and minor salivary glands.^2^ Salivary tumours are uncommon in children, but a greater proportion (20–30 per cent) of them are malignant (usually low-grade mucoepidermoid carcinomas).

Salivary gland tumours present a diverse range of histological and clinical behaviours. The rarity of these tumours combined with the diverse histology means that there is a lack of studies that can be used to provide strong recommendations for each individual histologic subtype of salivary tumour. The World Health Organization (WHO) classification has been modified on a number of occasions, the last being in 2005.^3^ A list of the more common adenomas and carcinomas is given in Table 1. Each histologic subtype is supposedly a unique entity in itself, but this notion has to be accepted with caution. Salivary gland neoplasms are generally slow growing lesions and patients have to be followed up for 10 years or more before one is confident of the natural history of the histological entity. In most instances, this information is not available. At present the unique clinical behaviour of many of the new subtypes is still to be identified.

**Table I:** Who classification of salivary gland tumours 2005^3^

Malignant epithelial tumours Acinic cell carcinoma Mucoepidermoid carcinoma Adenoid cystic carcinoma Polymorphous low-grade adenocarcinoma Epithelial myoepithelial carcinoma Clear cell carcinoma, not otherwise specified Basal cell adenocarcinoma Sebaceous carcinoma Sebaceous lymphadenocarcinoma Cystadenocarcinoma Low-grade cribriform cystadenocarcinoma Mucinous adenocarcinoma Oncocytic carcinoma Salivary duct carcinoma Squamous cell carcinoma Undifferentiated carcinoma Small cell carcinoma Large cell carcinoma Lymphoepithelial carcinoma Adenocarcinoma, not otherwise specified Carcinoma ex pleomorphic adenoma malignant mixed tumour Myoepithelial carcinoma Soft tissue tumours Haemangioma Haematolymphoid tumours Hodgkin lymphoma Metastasising pleomorphic adenoma Diffuse large B-cell lymphoma Extranodal marginal zone B cell lymphoma Secondary tumours Soft tissue Haematopoetic Benign epithelial tumours Pleomorphic adenoma Myoepithelioma Basal cell adenoma Warthin's tumour (adenolymphoma) Oncocytoma Cystadenoma Papillary cystadenoma Mucinous cystadenoma Keratocystoma Canalicular adenoma Sialadenoma papilliferum Sebaceous adenoma Sialoblastoma Lymphadenoma Benign papilloma (intraductal/inverted ductal/ductal)

WHO = World Health Organization

Carcinomas are often further classified as high grade, low grade or mixed, the latter inferring a variable behaviour depending on the histological picture. Except in the case of mucoepidermoid tumours, the clinicopathological correlation has proved unreliable. It should be recognised that the clinical behaviour rather than the histology of a tumour provides a better treatment guide and it is important to consider clinical factors in addition to histology and grade when planning treatment.^4^

## Clinical presentation

In general, salivary tumours are present in two forms: a simple palpable lump (well-defined, discrete and mobile) or a lump with significant accompanying symptoms (pain, rapid growth, fixity to surrounding structures, nerve involvement or neck metastasis). The latter features are all suggestive of malignancy. Both should be seen in a rapid access neck lump clinic ideally to have the appropriate assessments and management plans formulated.

## Assessment and staging

A third of malignant tumours have an indolent nature and may be clinically indistinguishable from benign lesions. Open biopsy is not encouraged in apparently benign lesions as it carries a theoretical risk of seeding, but it sometimes has a role in the frankly malignant lesion (open or core biopsy) especially when radical surgery is being contemplated. As indolent lesions may masquerade as benign lumps the definitive histology sometimes may not be available until after surgical resection. Diagnosis and management of these tumours is therefore based on the clinical presentation, imaging and cytology and/or histology results.

### Fine needle aspiration cytology (FNAC) and core biopsy

This is the primary diagnostic tool for salivary gland lesions (parotid, submandibular and minor salivary), and has additional value if examined by a cytopathologist or pathologist experienced in the diagnosis of salivary gland disease. This can distinguish malignant from benign disease in 90 per cent of cases.^5^ However, it is essential to ensure that fine needle aspiration results are interpreted in the context of all clinical information.

### Imaging considerations

Ultrasound by a skilled head and neck radiologist is an essential tool as part of initial assessment and provides excellent information about the primary tumour as well as cervical lymph node status.^6^ It can be combined with FNAC and in experienced hands, helps distinguish benign from malignant lesions in about 80 per cent of cases.

**Recommendation**

• Ultrasound guided FNAC is recommended for all salivary tumours and cytology should be reported by an expert histopathologist (R)

Non-homogeneous, muscle infiltration or suspicious regional lymph node appearances on cross-sectional imaging (computed tomography (CT) or magnetic resonance imaging (MRI)) are suggestive of malignancy. However, its main role is to determine size, position and relationship to adjacent structures. Computed tomography imaging is useful in proven malignancy to exclude distant metastases which carry a poor prognosis.

### Open biopsy

This should be avoided in major salivary gland lesions due to a risk of spillage unless the lesion appears frankly malignant and no cytological diagnosis has been made. In this instance, histological confirmation may inform planning of a more radical surgical approach. Histology may still be obtained by the use of ultrasound guided core biopsy specimens rather than an open biopsy. For minor salivary glands, open biopsy is permissible, but where possible should be undertaken by a dermatological punch.

### Frozen section

Accurate diagnosis is often difficult and false negative rates are significant therefore it is essential that if frozen section is being considered it must be done by an expert pathologist.^7^ On some occasions pre-operative cytology and/or histology may remain unclear and therefore the frozen section may have a role in parotid surgery. It is important not to breach the tumour capsule during parotid surgery and a partial parotidectomy specimen should be sent for the frozen section which may help determine the presence of malignancy and therefore help inform a decision regarding proceeding to radical surgery. This may be preferred rather than waiting for results of a partial parotidectomy as completion parotidectomy at a later stage carries a significant morbidity, especially with regards to facial nerve function.

### Staging

The Tumour–Node–Metastases (7) system staging for salivary gland primary tumour is shown in Table II.

**Table II:** T-staging for salivary gland tumours

Tx	Primary tumour cannot be assessed
T0	No evidence of primary tumour
T1	Tumour ≤2 cm in greatest dimension without extraparenchymal extension*
T2	Tumour >2 cm but ≤4 cm in greatest dimension without extraparenchymal extension*
T3	Tumour >4 cm and/or tumour having extraparenchymal extension*
T4a	Tumour invades skin, mandible, ear canal and/or facial nerve
T4b	Tumour invades skull base and/or pterygoid plates and/or encases carotid artery

* Extraparenchymal extension is clinical or macroscopic evidence of invasion of soft tissues. Microscopic evidence alone does not constitute extraparenchymal extension for classification purposes.

The staging of metastatic neck nodes for salivary gland cancer is similar to that for other metastatic disease.

## Management

### Submandibular gland

#### Benign tumours

The submandibular gland should be excised in a supracapsular plane. A wide dissection of local tissues is not required.

#### Malignant tumours

Historical survival rates in submandibular gland cancer are lower than those achieved in parotid or minor salivary gland malignancy.^8^^,^^9^ This has been attributed to the absence of a pre-treatment malignant diagnosis and therefore performance of conservative resection. It is important that if a neoplasm is suspected or a firm supposedly fibrotic submandibular gland encountered then every effort should be made to establish whether it is benign or malignant prior to surgery.

##### Surgical management of the primary tumour

Wide excision is appropriate for tumours confined to the gland combined with some form of neck dissection. Some argue in favour of a wider resection for adenoid cystic tumours but even with radical surgery, it is frequently difficult to obtain adequate surgical margins.^10^ The advice for radical surgery in submandibular malignancy is at variance with recommendations for the preservation of the uninvolved facial nerve in parotid disease. Clinically high-grade tumours should be treated aggressively with excision of the gland plus a 2 cm margin of apparently healthy tissue. Resection of involved nerves with microscopic negative margins is desirable. Large infiltrative tumours with bony involvement are treated with composite resection of tumour, adjacent soft tissue cuff and bony resection as appropriate.

##### Surgical management of the neck metastases

In the N0 neck, patients should undergo clearance of nodes with a selective neck dissection (levels 1 and 2A). Clinically high-grade tumours or tumours with suspicious MRI appearances should have an elective selective dissection (levels 1–3).

The following histological types have higher risk of metastasis: high-grade mucoepidermoid carcinoma, squamous cell carcinoma (SCC), anaplastic tumours and carcinoma ex pleomorphic adenoma. Carcinoma ex pleomorphic adenoma has been redefined and some types act as benign tumours.^11^ It is the frankly malignant variety that carries the risk of metastases. Patients with clinically confirmed neck metastasis (N+) should have a neck dissection, the extent of which will be based on disease stage and location.

##### Primary radiotherapy (RT)

Primary RT may be applicable in inoperable tumours where palliation can be achieved.^12^ The role of heavy ions such as proton and carbon ion therapy is being explored and is as yet unresolved.

##### Post-operative RT

‘The 4 cm rule’: survival is significantly worse in tumours greater than 4 cm in diameter.^12^ With increasing size the risk of occult metastasis is greater and tumour size is a major determinant of distant metastasis. Tumours greater than 4 cm in size fall into the class of high risk or complex tumours, and adjuvant RT is advised. Post-operative RT should be commenced within six weeks of surgery.

Indications for post-operative RT:

• High-grade or advanced stage tumours (>4 cm) with a high risk of local recurrence
• Residual neck disease or microscopic extracapsular spread from lymph nodes
• Following surgery for recurrent disease
• Adenoid cystic carcinomas (ACC).

##### Surveillance

Following surgery alone or surgery followed by RT careful surveillance is required. Ultrasound offers an accurate method of detecting recurrence but a baseline MRI three months following treatment is useful for comparison.

**Recommendation**

• Adjuvant RT following surgery is recommended for all malignant submandibular tumours except in cases of small, low-grade tumours that have been completely excised (R)

### Parotid gland

#### Benign tumours

Traditional management of benign parotid tumours is by dissection of the facial nerve leading to a superficial or total parotidectomy. There is currently no agreement in the literature as to the extent of resection to obtain an adequate margin in benign tumours.^13^ There is increasing recognition that operations less than the traditional procedures (extracapsular dissection, partial parotidectomy and even endoscopically assisted parotidectomy) are as effective in selected patients.^14^ It is preferable that these procedures should be performed by expert surgeons in appropriately selected cases, such as small tumours confined to the superficial lobe. A ‘lumpectomy’ procedure should not be done due to high recurrence rates. As the facial nerve not infrequently is very close to the tumour (especially in larger tumours) careful dissection avoiding tumour rupture is important. Tumour spillage carries an increase in the rate of recurrence over a prolonged period and therefore long-term follow-up is recommended in such cases.^15^ Adjuvant RT for such cases should be discussed in a multidisciplinary team (MDT) setting, but the use of RT in these cases is controversial and is generally not recommended especially in younger patients due to the risk of radiation-induced tumours.

#### Recurrent benign parotid tumours

These will usually be treated surgically. Careful pre-operative ultrasound marking may be helpful. A widefield removal of tissue in the parotid bed with preservation of the facial nerve is recommended. The patient should be discussed in the MDT for the suitability of post-operative RT to reduce re-recurrence.

**Recommendations**

• For benign parotid tumours complete excision of the tumour should be performed and offers good cure rates (R)
• In the event of intra-operative tumour spillage, most cases need long-term follow-up for clinical observation only. These should be discussed in the MDT to discuss the merits of adjuvant RT (G)

#### Malignant tumours

##### Surgical management of the primary tumour

In carcinoma, surgery is the treatment of choice with management tailored to the individual case.^16^ A conservative parotidectomy should be performed with preservation of the functioning facial nerve providing there is no tumour invasion. For small, low-grade superficial tumours a partial parotidectomy (superficial parotidectomy or wide local resection with an adequate margin of at least 1.5 cm) may suffice but otherwise a total conservative parotidectomy is advocated with resection of adjacent structures if necessary to achieve an en-bloc resection. Any part of the facial nerve not infiltrated by tumour should be preserved and a frozen section may be needed to determine nerve involvement. If the facial nerve is functional pre-operatively, then primary nerve grafting should be performed following radical resection. Adenoid cystic carcinoma characteristically has a diffuse pattern of spread and incomplete surgical clearance is the norm. A total parotidectomy with sacrifice of any part of any of the nerves overtly involved in tumour is desirable.

##### Management of the facial nerve in the context of parotid tumours

The facial nerve can be damaged as a sequelae of parotid surgery, either as a planned event when removing a malignant tumour or inadvertently. The damage can be a division of the nerve or can occur with the nerve intact, i.e. a neuropraxia. If the nerve is divided, it should be repaired as soon as possible, ideally acutely. Direct microsurgical repair without tension, or repair utilising a nerve graft, offer the best chance of a good recovery. A delay of more than a year in nerve repair has been shown to be an adverse factor in patient recovery. If a proximal nerve stump is not available or significant amounts of facial muscle have been removed, facial re-animation will require importation of a new muscle and nerve supply. The facial nerve can routinely be found and exposed in the mastoid segment of the temporal bone if the nerve cannot be found in the neck. Re-animation techniques can be one stage using either microsurgical importation of a free muscle transfer, or regional involving a temporalis myoplasty. Such techniques require substantial expertise and patients with significant facial paralysis should be referred to a service which can offer a full spectrum of reconstructive options regarding facial re-animation, including care of the eye.

**Recommendation**

• As a general principle, if the facial nerve function is normal pre-operatively then every attempt to preserve facial nerve function should be made during parotidectomy and if the facial nerve is divided intra-operatively then immediate microsurgical repair (with an interposition nerve graft if required) should be considered (G)

##### Surgical management of the neck metastases

The literature reports rates between 13 and 39 per cent of pathological neck node metastases in parotid cancer. Neck dissection should be performed in patients with clinical or radiological evidence of nodal disease.^17^

A prophylactic selective neck dissection should be considered for patients with high-stage and/or clinically high-grade tumours (i.e. adenocarcinoma, squamous and undifferentiated carcinomas, high-grade mucoepidermoid carcinoma and carcinoma ex pleomorphic adenoma).^18^^,^^19^ In addition, neck dissection provides a histological specimen which provides important prognostic information such as extracapsular spread which has been shown to be a poor prognostic indicator.

**Recommendation**

• Neck dissection is recommended in all cases of malignant parotid tumours except for low-grade small tumours (R)

##### Radiotherapy

Radiotherapy is effective in reducing the risk of recurrent benign tumours. It has application in high risk of recurrence pleomorphic adenoma cases, namely gross wound contamination and as an adjuvant therapy for treatment of multinodular recurrent disease.^15^

Adjuvant RT is appropriate for large tumours (>4 cm), recurrent disease, patients with incomplete or close margins, peri-neural invasion, extension beyond the gland, nodal disease, in metastatic disease and is increasingly the norm following treatment of ACC and high-grade tumours.^12^

**Recommendations**

• Where malignant parotid tumours lie in close proximity to the facial nerve there should be a low threshold for adjuvant RT (G)
• Adjuvant radiation should be considered in high-grade or large tumours or in cases where there is incomplete or close resection margin (R)
• Adjuvant radiation should be prescribed on the basis of clinical factors in addition to histology and grade, e.g. stage, pre-operative facial weakness, positive margins, peri-neural invasion and extracapsular spread (R)

#### Recurrent cancer

This requires careful evaluation of the patient with repeat imaging and a review of the histology from the initial excision. It will usually require more radical surgery with sacrifice of the facial nerve and overlying skin if there is suspicion of involvement by tumour. Super-radical resections of the skull base have not to date shown convincing evidence of improved survival. Consider chemotherapy and/or RT for palliation.

### Minor salivary glands

The natural history of intra-oral minor salivary gland tumours is similar to the parotid and submandibular glands. Outcome is worse for ‘hidden sites’, i.e larynx, nasopharynx and nose. The prognosis for these patients as with parotid and submandibular glands is related to stage of disease rather than the histology.

#### Benign tumours

Tumours of the palate can be safely resected at the subperiosteal level without removing palatal bone. Proven benign tumours in soft tissue can be removed by careful local dissection.

#### Malignant tumours

##### Surgical management of the primary tumour

Most cases are treated in a similar way to SCC, with en-bloc resection with depth of excision compatible with treatment of SCC to ensure adequate resection margins.^20^ Significant defects are repaired as appropriate.

##### Surgical management of the neck metastases

Therapeutic neck dissection is indicated for lymph node involvement. Elective neck dissection is indicated for high-stage and clinically high-grade disease such as high-grade adenocarcinoma, invasive carcinoma ex pleomorphic adenoma, SCC, high-grade mucoepidermoid and undifferentiated carcinoma.^18^

##### Radiotherapy

The following factors are indications for post-operative RT^12^:

• Microscopic residual disease
• Adenoid cystic tumours
• Aggressive undifferentiated tumours
• A‘4 cm rule’.

## Natural history and management principles for common salivary malignancies

### Acinic cell carcinomas

These tumours account for about 3 per cent of parotid tumours, where they occur most commonly. Peak incidence is in the fifth decade. Other features include:

• A variable histological pattern, multifocal origin and occasionally bilateral location
• Determinate survival rates of 90 per cent at five years and 55 per cent at 20 years^9^
• Lymph node metastatic rate of approximately 10 per cent.

Most acinic cell cancers are low grade (approximately 80 per cent). Small indolent peripheral lesions can be managed by less than a total parotidectomy. In low-grade acinic cell cancers, adjuvant RT following complete excision may not confer survival benefit and therefore the role of adjuvant RT should be considered carefully in such cases.^21^ Total parotidectomy with preservation of uninvolved nerves is recommended. Elective neck dissection is usually not indicated.

### Mucoepidermoid tumour

These tumours have variable malignant potential with low-grade lesions following an indolent course. Histologically high-grade lesions have a natural history similar to SCC. The histological grade correlates with several prognostic factors including presence of lymphatic spread and survival.^9^^,^^22^ The key features are detailed below:

• Most common major salivary gland tumour (4–9 per cent) with over 90 per cent occurring in the parotid but overall more frequent in minor salivary glands
• Most common malignant salivary gland tumour in children and usually presents in its indolent form
• Highest incidence third to fifth decade with no difference in gender incidence
• In the parotid, it is almost always in the superficial lobe
• Histological division into low, intermediate and high-grade correlates with prognosis, although ‘low-grade’ tumours can on occasion be aggressive^22^
• Five-year survival varies between 86 per cent for low-grade and 22 per cent for high-grade tumours. Peri-neural and lymphovascular invasion is not uncommon in these tumours
• Incidence of lymph node metastases is 40 per cent in intermediate and high-grade tumours
• Small low-grade tumours can be removed by less than a total parotidectomy and bigger ones (>2 cm) will frequently be in close contact with the facial nerve and the aforementioned advice pertains regarding adjuvant RT
• Adjuvant RT indicated for high-grade tumours.

**Recommendation**

• In cases of mucoepidermoid carcinoma, the histologic grade is an important factor correlating to outcome and should be considered when planning treatment (G)

### Adenoid cystic carcinoma

This is the most common salivary gland malignancy (20–25 per cent of all malignant salivary gland neoplasms), occurs at mucosal sites more frequently than in major salivary glands, and accounts for 2–6 per cent of parotid tumours and approximately 15 per cent of submandibular tumours. It is characterised by:

• Slow, pervasive growth and a high incidence of peri-neural infiltration. Relapsing neuralgic type pain can be a feature of early hidden disease. Patients can be ‘labelled’ as having atypical facial pain with consequent diagnostic delay
• Variable histologic appearance but difficult to correlate with clinical behaviour although some report cribriform pattern to have better prognosis than tubular or solid pattern tumours.

Treatment should involve wide local excision with preservation of uninvolved major nerves. Adjuvant post-operative RT is often indicated.^10^ Stage I and II cancers can be cured although the survival curve never flattens even after 20 years. Patient with stage III and IV diseases have a poor prognosis with low survival rates at 10 years. Slow growth rate makes five-year survival rates unreliable marker of cure. Only 20 per cent of patients with pulmonary metastases survive more than five years.

### Adenocarcinoma

This uncommon tumour is most frequently (90 per cent) found in the parotid gland. It is characterised by:

• Equivalent gender incidence, affecting any age and is one of the commonest tumours seen in children
• Histologic appearances varying between low-grade well-differentiated papillary or mucinous patterns to high-grade, undifferentiated lesions
• Distant metastatic incidence rates of 40 per cent for high-grade tumours, directly related to survival rates
• A 75 and 19 per cent five-year survival for low-grade and high-grade tumours, respectively.

Treatment is by wide local resection with elective neck dissection and adjuvant RT for clinically high-grade tumours.

### Malignant mixed tumour (carcinoma ex pleomorphic adenoma)

Carcinoma ex pleomorphic adenoma is a broad category of carcinomas of the salivary glands.^11^ The name is probably a misnomer for only a minority of malignant mixed tumours arise from pleomorphic adenomata. These are typically tumours with a history of multiple recurrences with surgery and RT. The remainder are probably not a homogeneous group of tumours and may occur *de novo* rather than following a malignant generation of pleomorphic adenoma. The frequency varies between 2 and 5 per cent.

Different patterns of malignant change can occur in pleomorphic adenoma of which the most commonly encountered is carcinoma ex pleomorphic adenoma, the other types being a true malignant mixed tumour and metastasising pleomorphic adenoma.

The tumours are classed as in situ carcinoma, non-invasive (intra-capsular including in situ carcinoma) minimally invasive (<1.5 mm) and invasive.^11^ The clinical behaviour of early or minimally invasive tumours is similar to that of pleomorphic salivary adenoma. Clinical malignant behaviour is associated with only the malignant foci that extend beyond the capsule of the tumour and these tumours tend to be high grade with a high incidence of haematogenous metastasis. Cure rates at 5, 10 and 15 years are 40, 24 and 19 per cent, respectively.

### Squamous cell carcinoma

This rare primary tumour is often mistaken for either a high-grade mucoepidermoid lesion or metastasis from another primary site; however, it is commonly metastases from a skin cancer. Features of this cancer include:

• Male to female incidence ratio 2:1
• Tendency to occur in the elderly (seventh decade)
• Metastatic disease presents initially as a discrete lump in the parotid however unlike salivary neoplasms SCC has a propensity for early extracapsular extension. In the parotid, this threatens local structures and prompt surgical intervention should be the rule. A few weeks' delay can make a significant difference to the complexity of surgery
• A poor prognosis and should be treated as high-grade mucoepidermoid lesions.

Radical resection with adjuvant RT offers the best form of management.

#### Key points

• There is a limited amount of clinical evidence for the management of salivary gland tumours
• Salivary gland tumours exhibit a diverse range of histological type and clinical behaviour
• Salivary gland malignancies are rare
• Investigations are essential to help tailor appropriate treatment and should include an FNA reported by an expert pathologist
• The majority of tumours will be treated by surgery, the extent of which should be tailored to the size, clinical and histological type of tumour
• As a general principle in cases where there is normal facial nerve function then the facial nerve should be preserved during surgical treatment
• Adjuvant radiotherapy should be considered in malignant cases with adverse clinical or histological features
• Elective treatment of the neck will be required in the majority of malignant tumours.
